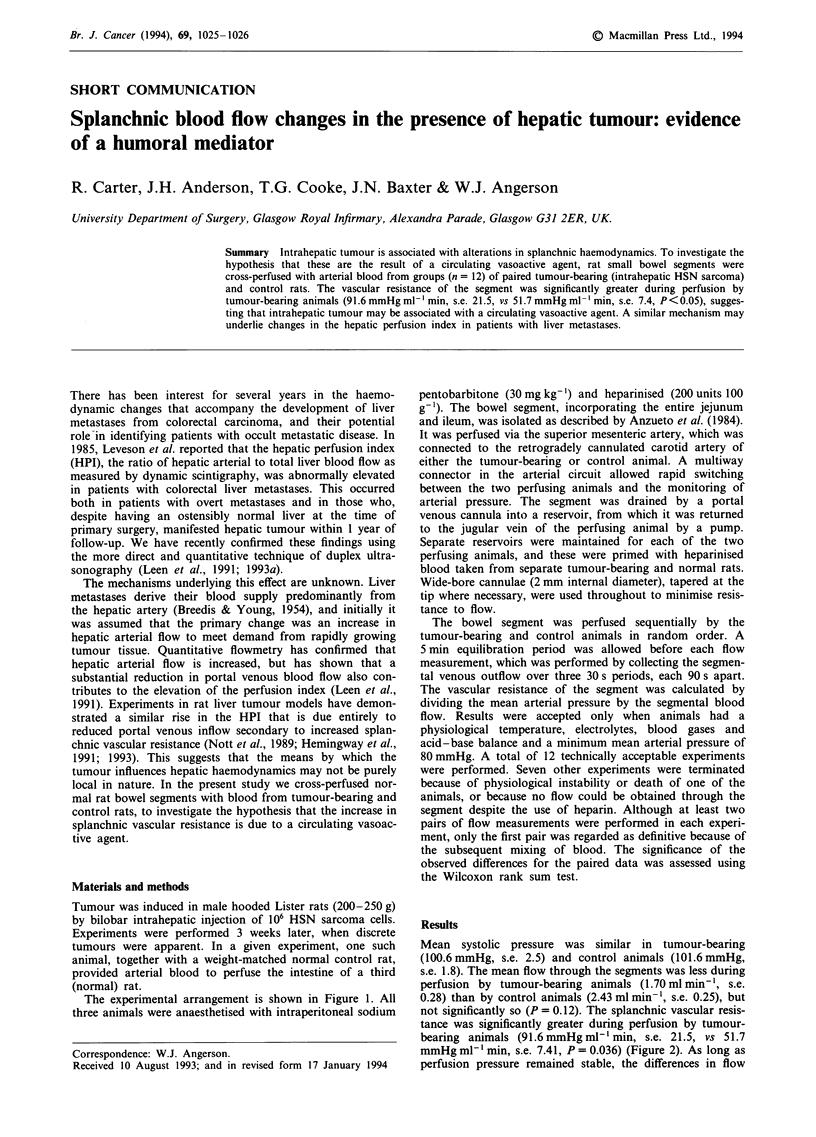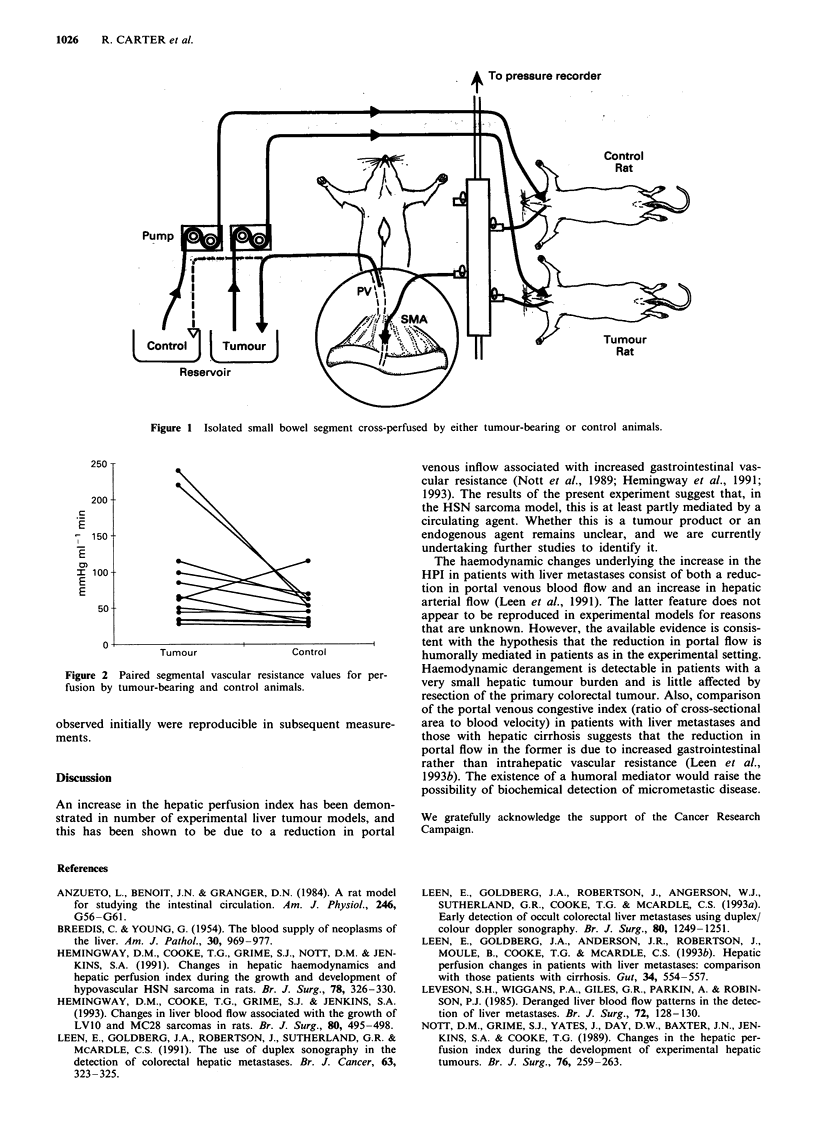# Splanchnic blood flow changes in the presence of hepatic tumour: evidence of a humoral mediator.

**DOI:** 10.1038/bjc.1994.201

**Published:** 1994-06

**Authors:** R. Carter, J. H. Anderson, T. G. Cooke, J. N. Baxter, W. J. Angerson

**Affiliations:** University Department of Surgery, Glasgow Royal Infirmary, UK.

## Abstract

Intrahepatic tumour is associated with alterations in splanchnic haemodynamics. To investigate the hypothesis that these are the result of a circulating vasoactive agent, rat small bowel segments were cross-perfused with arterial blood from groups (n = 12) of paired tumour-bearing (intrahepatic HSN sarcoma) and control rats. The vascular resistance of the segment was significantly greater during perfusion by tumour-bearing animals (91.6 mmHg ml-1 min, s.e. 21.5, vs 51.7 mmHg ml-1 min, s.e. 7.4, P < 0.05), suggesting that intrahepatic tumour may be associated with a circulating vasoactive agent. A similar mechanism may underlie changes in the hepatic perfusion index in patients with liver metastases.


Br. J. Cancer (1994), 69, 1025–1026 © Macmillan Press Ltd., 1994

SHORT COMMUNICATION

### Splanchnic blood flow changes in the presence of hepatic tumour: evidence of a humoral mediator

R. Carter, J.H. Anderson, T.G. Cooke, J.N. Baxter & W.J. Angerson

*University Department of Surgery, Glasgow Royal Infirmary, Alexandra Parade, Glasgow G31 2ER, UK.*

**Summary** Intrahepatic tumour is associated with alterations in splanchnic haemodynamics. To investigate the hypothesis that these are the result of a circulating vasoactive agent, rat small bowel segments were cross-perfused with arterial blood from groups ($n = 12$) of paired tumour-bearing (intrahepatic HSN sarcoma) and control rats. The vascular resistance of the segment was significantly greater during perfusion by tumour-bearing animals (91.6 mmHg ml$^{-1}$ min, s.e. 21.5, *vs* 51.7 mmHg ml$^{-1}$ min, s.e. 7.4, $P<0.05$), suggesting that intrahepatic tumour may be associated with a circulating vasoactive agent. A similar mechanism may underlie changes in the hepatic perfusion index in patients with liver metastases.

There has been interest for several years in the haemodynamic changes that accompany the development of liver metastases from colorectal carcinoma, and their potential role in identifying patients with occult metastatic disease. In 1985, Leveson *et al.* reported that the hepatic perfusion index (HPI), the ratio of hepatic arterial to total liver blood flow as measured by dynamic scintigraphy, was abnormally elevated in patients with colorectal liver metastases. This occurred both in patients with overt metastases and in those who, despite having an ostensibly normal liver at the time of primary surgery, manifested hepatic tumour within 1 year of follow-up. We have recently confirmed these findings using the more direct and quantitative technique of duplex ultrasonography (Leen *et al.*, 1991; 1993*a*).

The mechanisms underlying this effect are unknown. Liver metastases derive their blood supply predominantly from the hepatic artery (Breedis & Young, 1954), and initially it was assumed that the primary change was an increase in hepatic arterial flow to meet demand from rapidly growing tumour tissue. Quantitative flowmetry has confirmed that hepatic arterial flow is increased, but has shown that a substantial reduction in portal venous blood flow also contributes to the elevation of the perfusion index (Leen *et al.*, 1991). Experiments in rat liver tumour models have demonstrated a similar rise in the HPI that is due entirely to reduced portal venous inflow secondary to increased splanchnic vascular resistance (Nott *et al.*, 1989; Hemingway *et al.*, 1991; 1993). This suggests that the means by which the tumour influences hepatic haemodynamics may not be purely local in nature. In the present study we cross-perfused normal rat bowel segments with blood from tumour-bearing and control rats, to investigate the hypothesis that the increase in splanchnic vascular resistance is due to a circulating vasoactive agent.

### Materials and methods

Tumour was induced in male hooded Lister rats (200–250 g) by bilobar intrahepatic injection of $10^6$ HSN sarcoma cells. Experiments were performed 3 weeks later, when discrete tumours were apparent. In a given experiment, one such animal, together with a weight-matched normal control rat, provided arterial blood to perfuse the intestine of a third (normal) rat.

The experimental arrangement is shown in Figure 1. All three animals were anaesthetised with intraperitoneal sodium pentobarbitone (30 mg kg$^{-1}$) and heparinised (200 units 100 g$^{-1}$). The bowel segment, incorporating the entire jejunum and ileum, was isolated as described by Anzueto *et al.* (1984). It was perfused via the superior mesenteric artery, which was connected to the retrogradely cannulated carotid artery of either the tumour-bearing or control animal. A multiway connector in the arterial circuit allowed rapid switching between the two perfusing animals and the monitoring of arterial pressure. The segment was drained by a portal venous cannula into a reservoir, from which it was returned to the jugular vein of the perfusing animal by a pump. Separate reservoirs were maintained for each of the two perfusing animals, and these were primed with heparinised blood taken from separate tumour-bearing and normal rats. Wide-bore cannulae (2 mm internal diameter), tapered at the tip where necessary, were used throughout to minimise resistance to flow.

The bowel segment was perfused sequentially by the tumour-bearing and control animals in random order. A 5 min equilibration period was allowed before each flow measurement, which was performed by collecting the segmental venous outflow over three 30 s periods, each 90 s apart. The vascular resistance of the segment was calculated by dividing the mean arterial pressure by the segmental blood flow. Results were accepted only when animals had a physiological temperature, electrolytes, blood gases and acid–base balance and a minimum mean arterial pressure of 80 mmHg. A total of 12 technically acceptable experiments were performed. Seven other experiments were terminated because of physiological instability or death of one of the animals, or because no flow could be obtained through the segment despite the use of heparin. Although at least two pairs of flow measurements were performed in each experiment, only the first pair was regarded as definitive because of the subsequent mixing of blood. The significance of the observed differences for the paired data was assessed using the Wilcoxon rank sum test.

### Results

Mean systolic pressure was similar in tumour-bearing (100.6 mmHg, s.e. 2.5) and control animals (101.6 mmHg, s.e. 1.8). The mean flow through the segments was less during perfusion by tumour-bearing animals (1.70 ml min$^{-1}$, s.e. 0.28) than by control animals (2.43 ml min$^{-1}$, s.e. 0.25), but not significantly so ($P = 0.12$). The splanchnic vascular resistance was significantly greater during perfusion by tumour-bearing animals (91.6 mmHg ml$^{-1}$ min, s.e. 21.5, *vs* 51.7 mmHg ml$^{-1}$ min, s.e. 7.41, $P = 0.036$) (Figure 2). As long as perfusion pressure remained stable, the differences in flow

Correspondence: W.J. Angerson.
Received 10 August 1993; and in revised form 17 January 1994

**Figure 1** Isolated small bowel segment cross-perfused by either tumour-bearing or control animals.

**Figure 2** Paired segmental vascular resistance values for perfusion by tumour-bearing and control animals.

observed initially were reproducible in subsequent measurements.

### Discussion

An increase in the hepatic perfusion index has been demonstrated in number of experimental liver tumour models, and this has been shown to be due to a reduction in portal venous inflow associated with increased gastrointestinal vascular resistance (Nott *et al.*, 1989; Hemingway *et al.*, 1991; 1993). The results of the present experiment suggest that, in the HSN sarcoma model, this is at least partly mediated by a circulating agent. Whether this is a tumour product or an endogenous agent remains unclear, and we are currently undertaking further studies to identify it.

The haemodynamic changes underlying the increase in the HPI in patients with liver metastases consist of both a reduction in portal venous blood flow and an increase in hepatic arterial flow (Leen *et al.*, 1991). The latter feature does not appear to be reproduced in experimental models for reasons that are unknown. However, the available evidence is consistent with the hypothesis that the reduction in portal flow is humorally mediated in patients as in the experimental setting. Haemodynamic derangement is detectable in patients with a very small hepatic tumour burden and is little affected by resection of the primary colorectal tumour. Also, comparison of the portal venous congestive index (ratio of cross-sectional area to blood velocity) in patients with liver metastases and those with hepatic cirrhosis suggests that the reduction in portal flow in the former is due to increased gastrointestinal rather than intrahepatic vascular resistance (Leen *et al.*, 1993*b*). The existence of a humoral mediator would raise the possibility of biochemical detection of micrometastic disease.

We gratefully acknowledge the support of the Cancer Research Campaign.

### References

ANZUETO, L., BENOIT, J.N. & GRANGER, D.N. (1984). A rat model for studying the intestinal circulation. *Am. J. Physiol.*, **246,** G56–G61.

BREEDIS, C. & YOUNG, G. (1954). The blood supply of neoplasms of the liver. *Am. J. Pathol.*, **30,** 969–977.

HEMINGWAY, D.M., COOKE, T.G., GRIME, S.J., NOTT, D.M. & JENKINS, S.A. (1991). Changes in hepatic haemodynamics and hepatic perfusion index during the growth and development of hypovascular HSN sarcoma in rats. *Br. J. Surg.*, **78,** 326–330.

HEMINGWAY, D.M., COOKE, T.G., GRIME, S.J. & JENKINS, S.A. (1993). Changes in liver blood flow associated with the growth of LV10 and MC28 sarcomas in rats. *Br. J. Surg.*, **80,** 495–498.

LEEN, E., GOLDBERG, J.A., ROBERTSON, J., SUTHERLAND, G.R. & MCARDLE, C.S. (1991). The use of duplex sonography in the detection of colorectal hepatic metastases. *Br. J. Cancer*, **63,** 323–325.

LEEN, E., GOLDBERG, J.A., ROBERTSON, J., ANGERSON, W.J., SUTHERLAND, G.R., COOKE, T.G. & MCARDLE, C.S. (1993*a*). Early detection of occult colorectal liver metastases using duplex/colour doppler sonography. *Br. J. Surg.*, **80,** 1249–1251.

LEEN, E., GOLDBERG, J.A., ANDERSON, J.R., ROBERTSON, J., MOULE, B., COOKE, T.G. & MCARDLE, C.S. (1993*b*). Hepatic perfusion changes in patients with liver metastases: comparison with those patients with cirrhosis. *Gut*, **34,** 554–557.

LEVESON, S.H., WIGGANS, P.A., GILES, G.R., PARKIN, A. & ROBINSON, P.J. (1985). Deranged liver blood flow patterns in the detection of liver metastases. *Br. J. Surg.*, **72,** 128–130.

NOTT, D.M., GRIME, S.J., YATES, J., DAY, D.W., BAXTER, J.N., JENKINS, S.A. & COOKE, T.G. (1989). Changes in the hepatic perfusion index during the development of experimental hepatic tumours. *Br. J. Surg.*, **76,** 259–263.